# Impact of A Multidisciplinary Team Discussion for Genetic Lung Fibrosis

**DOI:** 10.1111/resp.70039

**Published:** 2025-03-26

**Authors:** Giovanni Franco, Ibrahima Ba, Nadia Nathan, Cécile Guerin, Albane Lassus, Caroline Kannengiesser, Antoine Froidure, Effrosyni Manali, Vincent Bunel, Philippe Bonniaud, Diane Bouvry, Marie Pierre Debray, Pierre Antoine Juge, Ralph Epaud, Camille Louvrier, Aurélie Plessier, Flore Sicre de Fontbrune, Lidwine Wémeau‐Stervinou, Sylvain Marchand Adam, Alexandre Chabrol, Arnaud Maurac, Laurent Savale, David Montani, Caroline Raynal, Marina Konyukh, Arthur Mageau, Bruno Crestani, Vincent Cottin, Alix de Becdelièvre, Raphaël Borie, Respifil and OrphaLung

**Affiliations:** ^1^ Université Paris Cité, Inserm, PHERE, Hôpital Bichat, AP‐HP, Service de Pneumologie A Centre Constitutif du Centre de Référence des Maladies Pulmonaires Rares, FHU APOLLO Paris France; ^2^ University of Milano Bicocca, School of Medicine and Surgery, UOC Pneumologia Fondazione IRCCS San Gerardo dei Tintori Monza Italy; ^3^ AP‐HP Service de Génétique Hôpital Bichat Paris France; ^4^ AP‐HP, Sorbonne Université, Pediatric Pulmonology Department and Reference Center for Rare Lung Disease RespiRare Armand Trousseau Hospital Paris France; ^5^ Pulmonology Department Cliniques Universitaires Saint‐Luc and Institut de Recherche Expérimentale et Clinique, UCLouvain Brussels Belgium; ^6^ 2nd Pulmonary Department, General University Hospital “Attikon”, Medical School National and Kapodistrian University of Athens Athens Greece; ^7^ Service de Pneumologie B et Transplantation Pulmonaire Hôpital Bichat, APHP, Inserm U1152, Université de Paris Paris France; ^8^ Centre de Référence Constitutif des Maladies Pulmonaires Rares de l'Adulte, Service de Pneumologie et Soins Intensifs Respiratoires Centre Hospitalo‐Universitaire de Dijon‐Bourgogne, Inserm 1231 CTM Dijon France; ^9^ Department of Pneumology, Centre Constitutif de Référence des Maladies Pulmonaires Rares, AP‐HP, Hôpital Avicenne, Bobigny, Unité mixte de recherche 1272 ‘Hypoxia and the Lung’, Institut national de la santé et de la recherche médicale (INSERM) Université Sorbonne Paris Nord Bobigny France; ^10^ APHP, Service de Radiologie Hôpital Bichat Paris France; ^11^ Service de Rhumatologie Hopital Bichat Paris France; ^12^ Service de pédiatrie, Centre Intercommunaux de Créteil Créteil France; ^13^ Département de Génétique Médicale, AP‐HP Sorbonne Université Hôpital Armand Trousseau Paris France; ^14^ Service d'Hépatologie, AP‐HP, Hôpital Beaujon, DMU DIGEST Centre de Référence des Maladies Vasculaires du Foie, FILFOIE, ERN RARE‐LIVER Clichy France; ^15^ Hematology Transplant Unit, French National Reference Center for Aplastic Anemia Hopital Saint Louis, APHP Paris France; ^16^ CHU Lille, Service de Pneumologie et Immuno‐Allergologie Centre de Référence des Maladies Pulmonaires Rares (Site Constitutif) Lille France; ^17^ Service de Pneumologie et d'explorations Fonctionnelles Respiratoires CHRU de Tours Tours France; ^18^ Service de Pneumologie Hôpital Foch, Suresnes Paris France; ^19^ Département de Pneumologie Hôpital Haut Lévèque, CHU de Bordeaux Pessac France; ^20^ Université Paris‐Saclay, AP‐HP, Service de Pneumologie et Soins Intensifs Respiratoires Hôpital de Bicêtre, DMU 5 Thorinno, Inserm UMR_S999 Le Kremlin‐Bicêtre France; ^21^ Laboratoire de Génétique Moléculaire Centre Hospitalier Universitaire et Université de Montpellier Montpellier France; ^22^ Department of Genetics, Henri Mondor University Hospital, APHP Univ Paris Est Creteil, INSERM, IMRB Créteil France; ^23^ Laboratoire de Biologie Médicale Multisites SeqOIA ‐ PFMG2025 Paris France; ^24^ Centre de référence des cytopénies Auto‐Immunes de l'adulte, Service de Médecine Interne Hôpital Henri Mondor, APHP, UPEC Créteil France; ^25^ Coordinating Reference Center for Rare Pulmonary Diseases, Louis Pradel Hospital University of Lyon, INRAE, UMR754, ERN‐LUNG Lyon France

**Keywords:** genetic disorder, interstitial pulmonary fibrosis, multidisciplinary discussion, surfactant related genes, telomerase related genes

## Abstract

**Background and Objective:**

Approximately 30% of individuals diagnosed with familial pulmonary fibrosis (FPF) exhibit a pathogenic variant upon genetic analysis. We established a genetic Multidisciplinary Discussion (geneMDD) aimed to enhance expertise in diagnosing and managing FPF. This study aimed at prospectively evaluating the impact of geneMDD on diagnosis and treatment in patients referred to geneMDD.

**Methods:**

In this prospective study, we enrolled all consecutive patients referred to the geneMDD. At each meeting, the impact of the meeting was questioned on the genetic conclusion, the pulmonary diagnosis, and the treatment.

**Results:**

A total of 115 patients were included. Before geneMDD, rare variants were detected in 82 out of 107 patients, among which 65 variants were classified as pathogenic/likely pathogenic. Following geneMDD, 2 pathogenic variants (3%) were reclassified as variants of uncertain significance (VUS) (*n* = 1) or benign (*n* = 1). Among the 17 variants initially classified as VUS, 2 (11.8%) were reclassified as likely pathogenic/pathogenic. The pulmonary diagnosis was confirmed for all patients (unclassifiable lung fibrosis was the more frequent diagnosis, *n* = 38, 33.0%). The therapeutic regimen was changed after geneMDD in 30 patients. Factors associated with therapeutic changes included the pulmonary diagnosis and presence of a pathogenic/likely pathogenic variant. In addition, the French health system allows offering whole genome sequencing (WGS) in patients with a first negative genetic analysis by NGS panel after discussion in geneMDD, but in total, since September 1st, 2021, WGS was negative for the four analysed families.

**Conclusion:**

This study suggests that geneMDD could influence the treatment of FPF patients.

## Abstract 2

GeneMDD could influence the treatment of FPF patients and might become the standard of care for patients with suspected or confirmed genetic ILD.

**Summary**

- GeneMDD could influence the treatment of FPF patients and might become the standard of care for patients with suspected or confirmed genetic ILD.

## 1 Introduction

Interstitial lung diseases (ILDs) constitute a vast and diverse category of pulmonary disorders characterised by varying degrees of lung inflammation and fibrosis, each presenting different prognoses and management strategies. Despite thorough analysis, diagnosing ILDs remains challenging, with the specific aetiology eluding identification in 10%–20% of cases [1]. This challenge arises from the shared respiratory clinic and frequent overlap of radiologic and/or histopathologic patterns among various diseases [2].

Since 2001, the American Thoracic Society (ATS) and the European Respiratory Society (ERS) emphasised the significance of a multidisciplinary discussion (MDD) based on the integration of clinical, radiologic, and pathologic data [3]. An ILD‐specific MDD should involve expert respiratory physicians, along with at least one radiologist and one histopathologist possessing specific expertise in ILD, unless biopsy or pathology specimens are not being reviewed [4, 5]. In challenging cases, the involvement of experienced rheumatologists and immunologists proves to be of utmost assistance [6]. The growing confidence in MDD as a highly accurate assessment of ILDs was underscored in the 2022 guidelines provided by the ATS, ERS, Japanese Respiratory Society (JRS), and Latin American Thoracic Association (ALAT) [7].

According to the ILD, up to 25% of the patients report a familial history of pulmonary fibrosis, though only 30% of individuals carry a likely pathogenic/pathogenic variant (LPV/PV) in telomere‐related genes (TRG), surfactant‐related genes (SRGs), or other rare genes [8, 9, 10, 11].

Disorders related to surfactant dysfunction, as genetic conditions, have been identified as fundamental causes of respiratory issues in both neonates and children, as well as adults. Managing these conditions necessitates collaboration with paediatricians with dedicated expertise [12]. In addition, LP/PV in TRG may be associated with extrapulmonary manifestations. For these reasons, genetic diagnosis in this field is notably challenging and demands a specialised expertise that may not be accessible in numerous ILD centres [8]. To provide the necessary expertise for the diagnosis, interpretation of genetic data, and treatment of patients suspected to have an inherited form of lung fibrosis, we established a web‐based genetic Multidisciplinary Discussion (geneMDD) in September 2016. The platform is dedicated to discussing all suspected or confirmed cases of inherited lung fibrosis [13]. As recently reported by the ERS taskforce on familial pulmonary fibrosis, the management of these patients should follow the current recommendations for the underlying ILD, although retrospective data may lead to discussions on specific points, especially concerning the diagnosis, immunosuppressants, or antifibrotics [14].

The aim of our study was to determine prospectively whether genetic analysis and geneMDD would alter the diagnosis, therapy, and need for additional investigations in patients. In addition, we report the results of whole genome sequencing (WGS) analyses as recently available as clinical care in France, within the French national program “*Plan France Médecine Génomique 2025*” (PFMG2025) [15], after a negative NGS panel analysis in cases of pulmonary fibrosis suspected to be of genetic origin.

## 2 Methods

### 2.1 geneMDD

The geneMDD established in September 2016 and later endorsed by the French OrphaLung network and the French RespiFil [16] organisations for rare respiratory diseases convenes monthly and includes at least one geneticist, one pneumologist with expertise in rare pulmonary diseases in adults, one paediatrician specialising in ILD, and a chest radiologist [13]. Additionally, a hepatologist and a haematologist may be present depending on the patient being discussed. Individuals with suspected inherited pulmonary fibrosis, regardless of age, are eligible for discussion, and genetic testing is not mandatory for a case to be discussed. The criteria for conducting a genetic analysis include familial pulmonary fibrosis, the presence of a specific syndrome indicative of heritable pulmonary fibrosis, such as short telomere syndrome, or cryptogenic pulmonary fibrosis before the age of 50 [14]. Cases involving deceased patients are discussed to address genetic counselling, considering the age at death as the age at presentation.

The meeting takes place online on a monthly basis. A specific form is sent before the meeting to the geneMDD coordinators for any patient who may be relevant for a genetic analysis.

During geneMDD, clinical data, chest CT scans, and lung histological patterns are reviewed and classified in accordance with the 2022 ATS/ERS/JRS/ALAT statement for IPF [7] and the 2013 ATS/ERS classification of idiopathic interstitial pneumonias [4].

When accessible, the geneMDD examines genetic and functional analysis findings. Genetic variants are classified according to the American College of Medical Genetics and Genomics and Association for Molecular Pathology (ACMG/AMP) [17], and the French *NGS‐diag* network working group recommendations. Additional analyses are recommended as needed, such as functional analyses [18, 19, 20], familial investigations, or extension of the genetic analyses. Genetic counselling can be recommended for both the affected patient and the relatives.

### 2.2 Inclusion Criteria

In this prospective study, we enrolled all consecutive patients, including asymptomatic relatives, referred to the geneMDD from September 1st, 2022 to February 29th, 2024. At each meeting, the impact of the meeting was specifically questioned on the genetic conclusion, the pulmonary diagnosis, and the treatment.

All patients signed informed consent for genetic analysis, including for research purposes. The clinical charts of the patients were collected on a standardised and anonymous form. This study was approved by the local ethics committee (CPP Ile de France 1, no. 0811760).

### 2.3 Whole Genome Sequencing

Since 2019, thanks to the national program whole genome sequencing is available as part of clinical care in France [15]. Inclusion criteria for PFMG2025 are (1) an indication for genetic analysis, (2) absence of a conclusive genetic diagnosis evidenced after a NGS panel analysis, (3) at least two individuals alive and available for analysis, regardless of the degree of relationship, with preference given to the closest degree of kinship. PFMG2025 indication should be discussed and validated during a specific gene MDD. Consents, samples, and sequencing are then specifically carried out in one of the two French sequencing platforms, SeqOIA (https://laboratoire‐seqoia.fr) or AURAGEN (https://www.auragen.fr). After analysis and interpretation, all results are discussed and presented during a dedicated gene MDD.

### 2.4 Statistical Analysis

Data are expressed as median (interquartile range) for quantitative variables and number (percentage) for categorical variables. We used Fisher's exact test to compare treatments before and after geneMDD. Because of separated data, we used Firth's penalised logistic regression to calculate odds ratios (ORs) and 95% confidence intervals (CIs) associated with patient characteristics for the risk of treatment modification recommendation. All *p* values were two‐sided, and statistical significance was defined as *p* < 0.05. Statistical analyses were performed with R v4.0.3.

## 3 Results

### 3.1 Patient Characteristics

**FIGURE 1:**
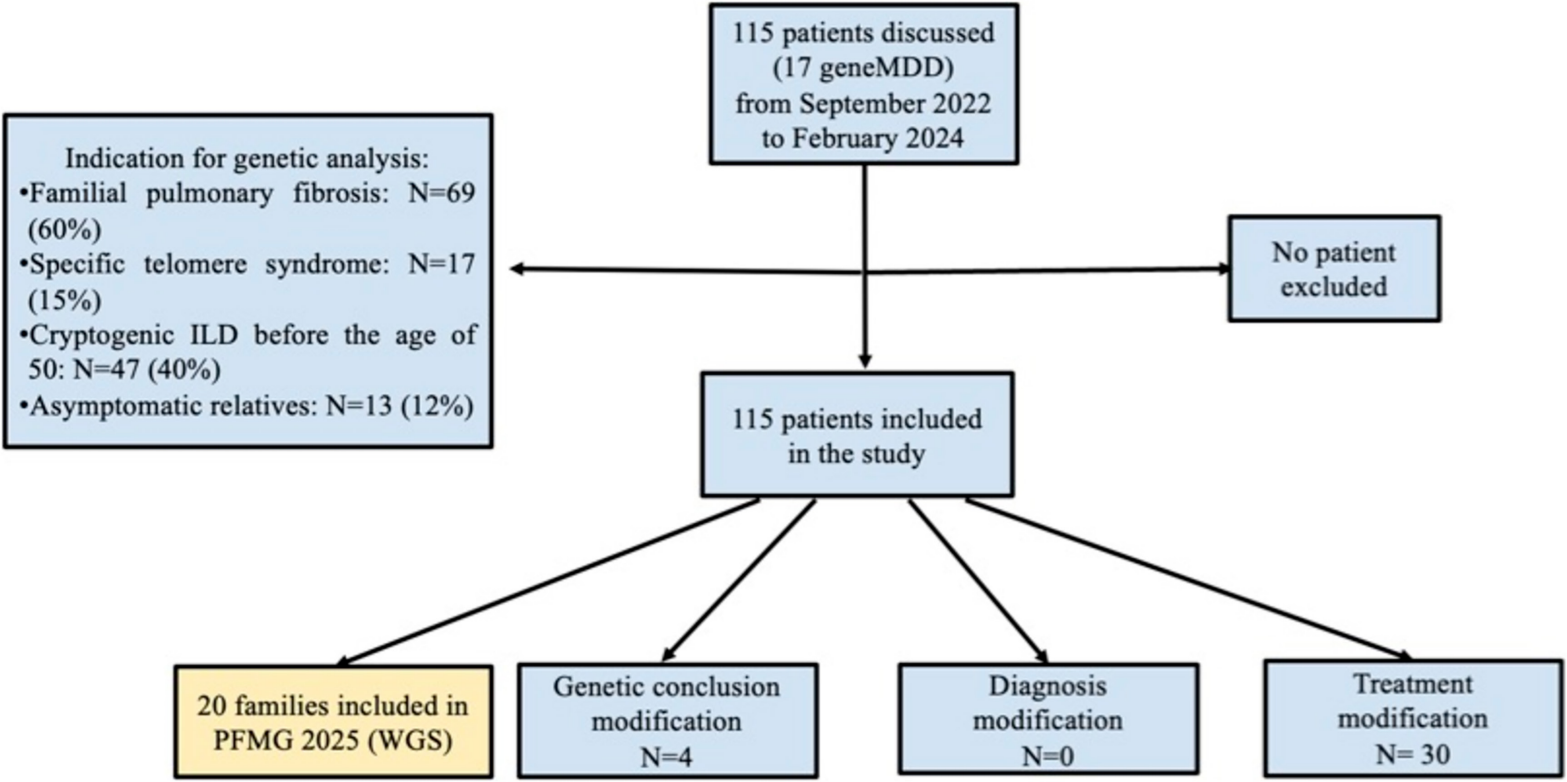
Flow‐chart of the study. geneMDD, genetic Multidisciplinary Discussion; ILD, interstitial lung diseases; PFMG, Plan France Médecine Génomique; WGS, whole genome sequencing.

From September 2022 to February 2024, the geneMDD took place 17 times with a participation of 28 different ILD centres from six different countries (Belgium, Denmark, France, Greece, Portugal, and Switzerland). In total, 115 patients (64 males—55.7%) from 110 different families were discussed (Figure 1, Table 1). The mean age of the patients was 53.8 ± 17.6 years [range 0–86]. At the time of the geneMDD, 10 patients (8.7%) were deceased. Patients could be discussed for several questions: interpretation of genetic analysis (*n* = 90, 78.3%), treatment options (*n* = 45, 39.1%), lung or liver transplantation (*n* = 14, 12.2%), and genetic counselling for relatives (*n* = 43, 37.4%).

**TABLE 1:** Patients' characteristics at the time of the meeting.

Characteristic	
Patients	115
Patient deceased at the time of geneMDD	10 (8.7%)
Asymptomatic relatives	13 (11.3%)
Patients with a family history of ILD	69 (60.0%)
Mean age at discussion	53.8 ± 17.6 [0–86]
Male/Female	64 (55.7%)/51 (44.3%)
Active or former smoker	40 (34.8%)
Pulmonary transplantation	2 (1.7%)
FVC % of predict	72 ± 25 [30–146]
FEV1% of predict (available for 60 patients)	69 ± 27 [26–142]
DLCO % of predict	44 ± 17 [11–82]

*Note*: Data are expressed as *n* (%) mean (± SD) [range].

Abbreviations: DLCO: diffusing capacity for carbon monoxide; FVC: forced vital capacity; geneMDD: genetic Multidisciplinary Discussion.

### 3.2 Genetic Analysis and Counselling

Indication for genetic testing was familial pulmonary fibrosis (*n* = 69, 60.0%), suspected short telomere syndrome (*n* = 17, 14.8%), cryptogenic ILD diagnosed before the age of 50 (*n* = 47, 40.1%), or asymptomatic relatives (*n* = 13, 12%). Genetic analyses results are summarised in Table 2. Genetic analyses were not available for 8 patients (7.0%) during the geneMDD assessment, with 4 undergoing the testing and analysis at the time of the meeting, and 4 yet to be conducted. Out of the 107 cases with a comprehensive genetic analysis, 104 patients underwent an NGS panel and 3 whole‐exome sequencing (WES).

**TABLE 2:** Genetic variant discussed during the geneMDD.

Genetic analysis		Patients with a family history of ILD, *N* (%)
Not performed, *n* (%)	4 (3.5%)	3 (4.3%)
Ongoing, *n* (%)	4 (3.5%)	2 (2.9%)
Negative for rare monoallelic or biallelic variants, *n* (%)	25 (23.4%)	18 (26.1%)
TRG, *n* (%)	54 (50.5%)	33 (47.8%)
*RTEL1*	23 (21.5%)	14 (20.3%)
*TERT*	18 (16.8%)	10 (14.5%)
*PARN*	5 (4.6%)	3 (4.3%)
*TERC*	3 (2.8%)	2 (2.9%)
*NHP2*	2 (1.9%)	1 (1.4%)
*DKC1*	1 (0.9%)	1 (1.4%)
*POT*	1 (0.9%)	1 (1.4%)
*ZCCHC8*	1 (0.9%)	1 (1.4%)
SRG, *n* (%)	22 (20.6%)	12 (17.4%)
*SFTPA1*	5 (4.7%)	4 (5.8%)
*SFTPA2*	5 (4.7%)	4 (5.8%)
*ABCA3*	5 (4.7%)	1 (1.4%)
*Homozygous*	3 (2.8%)	1 (1.4%)
*Heterozygous*	2 (1.9%)	0 (0.0%)
*NKX2‐1*	4 (3.7%)	2 (2.9%)
*SFTPC*	3 (2.8%)	1 (1.4%)
Other, *n* (%)	6 (5.6%)	1 (1.4%)
*CSF2RB*	1 (0.9%)	0 (0.0%)
*Homozygous*	1 (0.9%)	0 (0.0%)
*PCYT1A*	1 (0.9%)	0 (0.0%)
*RNF31*	1 (0.9%)	0 (0.0%)
*SCL34A2*	1 (0.9%)	0 (0.0%)
*Heterozygous*	1 (0.9%)	0 (0.0%)
*STING*	1 (0.9%)	0 (0.0%)
*USB1*	1 (0.9%)	1 (1.4%)

*Note*: Data are expressed as *n* (%).

Abbreviations: GeneMDD, genetic Multidisciplinary Discussion; SRGs, surfactant‐related genes; TRGs, telomere‐related genes.

Before the geneMDD, rare monoallelic or biallelic variants were detected in 82 out of the 107 (76.6%) patients analysed. These variants were consistent with either dominant or recessive inheritance patterns. Among these variants, 17 (20.7%) were classified as Variants of Uncertain Significance (VUS), while 65 (79.3%) were classified as pathogenic or likely pathogenic variants (PV and PLV) (Table 2).

After geneMDD, 63 variants (96.9%) initially classified as PV/LPV remained in the same classification. However, one *NHP2* frameshift variant was reclassified as VUS, and a missense variant in the *SFTPC* gene was reclassified as non‐pathogenic. The *NHP2* variant, initially interpreted as pathogenic, is a frameshift mutation. Family analysis revealed that the brother of the index case, who developed lymphoma, carries this variant, while his daughter does not. The 30‐year‐old daughter of the index case is also not a carrier, whereas his twin daughters both carry the variant. Notably, the father of the index case, who has pulmonary fibrosis, does not carry the *NHP2* variant. Given the lack of segregation between phenotype and genotype, and considering current knowledge, the *NHP2* variant has been reclassified as a class III variant (uncertain significance). Regarding the *SFTPC* variant, it was originally classified as pathogenic at the time of sequencing in 2012. However, recent data indicate that it occurs in the African population with a frequency of 1.65% and has been observed in the homozygous state in healthy individuals. Consequently, this variant can no longer be considered pathogenic based on the current evidence. Both patients underwent a pedigree study and a recent NGS panel analysis, which did not identify any other candidate genes.

Additionally, out of the 17 VUS, while 15 (88.2%) were still classified as VUS, 2 (11.8%) were reclassified as LPV/PV. One was a variant that induces the formation of a premature STOP codon in *RTEL1*, which was absent in the GnomAD database and was associated with short telomeres. This variant was reclassified as PV. The other variant affected a conserved amino acid in *ZCCHC8* and had not been described in the literature. This variant was subsequently found in another unrelated patient with a familial history of pulmonary fibrosis and cirrhosis in our laboratory. Following these considerations, it was therefore re‐interpreted as LPV.

Additional genetic evaluation was proposed for 32 patients (29.9%), which includes WES or targeted NGS for 15 patients (14.0%), functional analysis for 12 patients (11.2%), including telomere length measurement (*n* = 3, 2.8%), surfactant related gene analysis (*n* = 3, 2.8%) or interferon signature analysis (*n* = 3, 2.8%).

### 3.3 Whole Genome Sequencing (WGS)/PFMG2025

**FIGURE 2:**
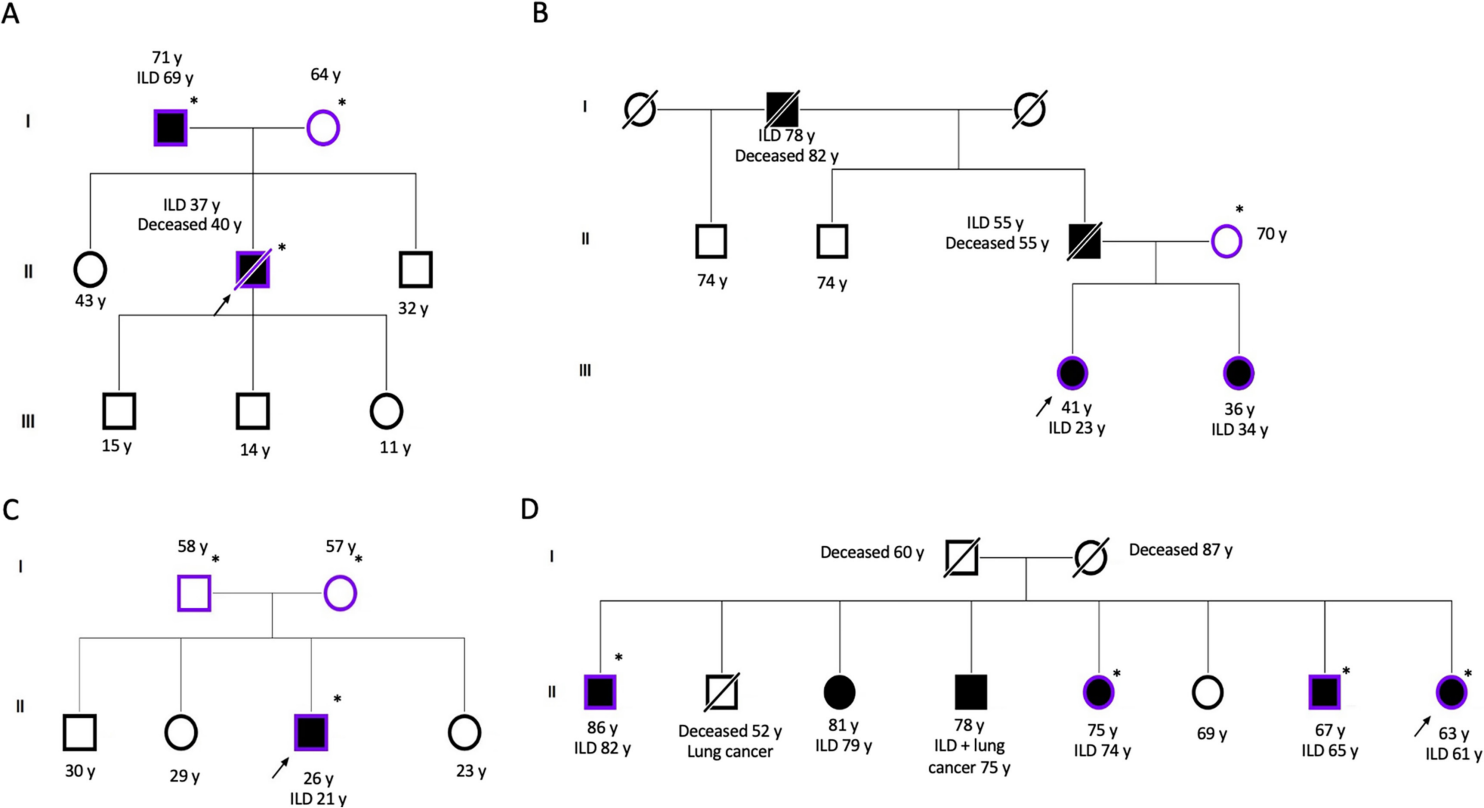
Pedigree of the families included in *Plan France Médecine Génomique*. Whole genome sequencing was performed in blood samples from 3 or 4 relatives in each family (indicated by *).

WGS began to be discussed in GeneMDD from September 2021. In total, from September 2021 to April 2024, 24 families were discussed for WGS. The indication was confirmed for 20 (83.3%) of them and referred to the dedicated platform: SeqOIA (*n* = 15) or AURAGEN (*n* = 5). DNA sampling is ongoing for 13 families (65%) and analysis is ongoing for 3 (15%). WGS analysis was available for 4 (20%) families (Figure 2) but did not reveal a LP/P variant responsible for pulmonary fibrosis for any of them.

### 3.4 Pulmonary Diagnosis and Treatment

All patients were basically discussed in local MDD, including expert clinician, radiologist, and pathologists with acknowledged recognition from the OrphaLung network for France (http://www.maladies‐pulmonaires‐rares.fr/). Before the geneMDD, the diagnosis was unclassifiable pulmonary fibrosis (*n* = 38, 33.0%), IPF (*n* = 37, 32.2%), NSIP (*n* = 11, 9.6%), PPFE (*n* = 6, 5.2%), cHP (*n* = 3, 2.6%), DIP (*n* = 3, 2.6%), alveolar proteinosis (*n* = 2, 1.7%), emphysema (*n* = 1, 0.9%), and pulmonary alveolar microlithiasis (*n* = 1, 0.9%). All patients with ILD had a fibrotic radiological pattern. Thirteen patients (11.3%) did not have ILD (Table S1). The pulmonary diagnosis was confirmed by the geneMDD for all the patients.

The genetic analyses modified the diagnostic or therapeutic proposal by local MDD for 13 patients prior to geneMDD. Before the geneMDD, among living patients (*n* = 105), 48 patients (45.7%) did not receive specific therapy, 35 patients (33.3%) received antifibrotic therapy (nintedanib *n* = 19 or pirfenidone *n* = 16), and 22 patients (21.0%) received immunomodulatory therapy, including prednisone (*n* = 12, 11.4%), mycophenolate mofetil (*n* = 4, 3.8%), azathioprine (*n* = 2, 1.9%), macrolides (*n* = 2, 1.9%), hydroxychloroquine (*n* = 1, 1.0%) or anakinra (*n* = 1, 1.0%).

### 3.5 The geneMDD Recommended a Treatment Change for 30 Patients (28.6%) (Table 3)

**TABLE 3:** Recommended modification of pharmacological treatment.

	Before MDD	MDD recommendation	*p*
No therapy	48 (45.7%)	33 (31.4%)	0.021
Antifibrotic	35 (33.3%)	54 (51.4%)	
Immunomodulatory	22 (21.0%)	16 (15.2%)	
Prednisone	12 (11.4%)	3 (2.9%)	
Mycophenolate mofetil	4 (3.8%)	4 (3.8%)	
Azathioprine	2 (1.9%)	1 (1.0%)	
Macrolide	2 (1.9%)	2 (1.9%)	
Hydroxychloroquine	1 (1.0%)	4 (3.8%)	
Anakinra	1 (1.0%)	1 (1.0%)	
Rituximab	0 (0.0%)	1 (1.0%)	
Target therapy	0 (0.0%)	2 (1.9%)	
Elexacaftor/Tezacaftor/Ivacaftor	0 (0.0%)	2 (1.9%)	

*Note*: Data are expressed as *n* (%).

Abbreviation: MDD, multidisciplinary discussion.

**FIGURE 3:**
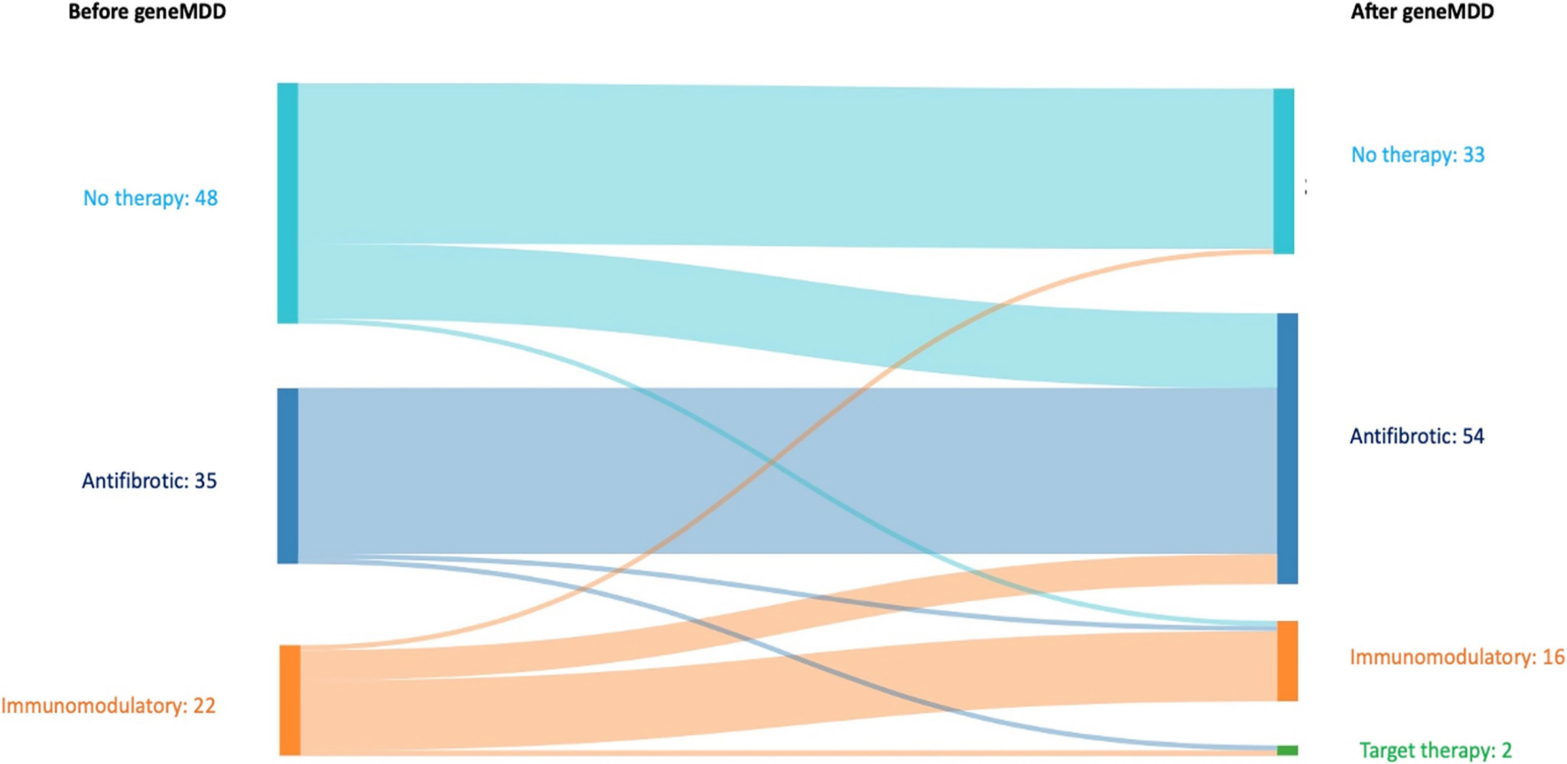
Sankey diagram summarising the changes in treatment recommended by the genetic multidisciplinary discussion.

Among the 48 patients without any therapy, geneMDD recommended treatment for 16 patients (33.3%): antifibrotics (*n* = 15) or immunomodulators (*n* = 1) (Figure 3). Among the 35 patients treated by antifibrotics, geneMDD confirmed the treatment for 33 patients (94.2%) and recommended a treatment change for 2 patients (immunomodulators *n* = 1, targeted therapy with a *CTFR* modulator for *ABCA3* pathogenic variants *n* = 1). Among the 22 patients on immunomodulators, geneMDD confirmed the therapy for 14 patients (63.6%) and recommended a treatment change for 7 patients, addition of an antifibrotic *n* = 6 or a repurposing targeted therapy with a *CFTR* modulator for *ABCA3* pathogenic variants *n* = 1, and to stop therapy in one patient (Figure 3). A survey was conducted after the geneMDD, and all centres reported that they applied the recommendations of the geneMDD.

Seventeen patients (16.2%) were specifically discussed for lung transplantation, one patient (1.0%) for liver transplantation, and 4 patients (3.8%) for lung‐liver combined transplantation. GeneMDD confirmed both the diagnosis and the indication for organ transplantation without contraindication due to available genetic, pulmonary, haematological, or hepatic data.

Lower age (*p* = 0.036), a non‐IPF ILD diagnosis (*p* = 0.009) and the presence of SRG (*p* = 0.005) and TRG (*p* = 0.012) variants were associated with therapeutic change recommendation in univariate and multivariable analysis (Table 4). Indeed, the geneMDD proposed to change therapy in 16 (43%) patients with unclassifiable ILD, 15 (32%) patients with a LP/P variant in TRG, and 8 (36%) in SRG.

**TABLE 4:** Univariate and multivariable Firth's penalised logistic regression analysis of factors associated with modified treatment recommendations.

Variable	Univariate analysis			Multivariable analysis		
	Odds ratio	CI	*p*	Odds ratio	CI	*p*
Age	0.98	0.96–0.99	0.036	0.97	0.94–1.00	0.074
IPF diagnosis	0.27	0.08–0.73	0.009	0.26	0.07–0.82	0.022
Genetic analysis						
Negative	Ref	Ref	Ref	Ref	Ref	Ref
SRG	9.57	1.87–96.7	0.005	18.1	2.83–214.9	0.001
TRG	6.41	1.44–60.9	0.012	11.4	1.97–136.2	0.004
Other	1.26	0.01–26.6	0.89	0.82	0.01–18.5	0.911

Abbreviations: IPF, idiopathic pulmonary fibrosis; SRGs, surfactant‐related genes; TRGs, telomere‐related genes.

## 4 Discussion

Our study is the first prospective study aimed to assess the impact of genetic analysis and multidisciplinary discussion on the diagnosis and treatment of patients with ILD of suspected genetic origin and showed that lower age, a non‐IPF ILD diagnosis, and the presence of SRG and TRG variants were associated with therapeutic change recommendation.

The multidisciplinary discussion for patients with suspected genetically originated ILD necessitates the involvement of specialists beyond the pulmonologist. With the growing number of genetic variants identified in ILD patients, genetic expertise is becoming more crucial in ILD centres. In our study, a rare variant within a single TRG was identified in 54 patients (50.5%). Patients carrying TRG pathogenic variants often exhibit haematological and hepatic conditions [18, 21, 22, 23], underscoring the need for the presence of a haematologist and hepatologist with specific expertise to comprehensively discuss these cases. Moreover, the geneMDD referred 21 patients (20.0%) for evaluation for a lung transplantation.

Multiple retrospective series have documented the outcomes of lung transplantation in patients with ILD and TRG pathogenic variants. Post‐transplant morbidity is higher in patients with telomere dysfunction and varies depending on the time elapsed since transplantation. However, lung transplantation improves survival and quality of life, and the associated complications are manageable [24]. While some studies highlight a distinct haematological risk and possible reduced survival in patients undergoing lung transplantation [25, 26], other studies suggest that patients with telomere‐mediated disease should not be excluded from lung transplantation, as transplant outcomes for survival and chronic lung allograft dysfunction do not differ based on gene variants or telomere length [27]. These observations once again underscore the importance of combining pulmonary diagnosis with genetic evaluation of the patient.

SRG abnormalities were the second most commonly identified genetic aetiology in patients discussed in geneMDD, emphasising the relevance of involving paediatricians in the discussion of patients with pathogenic variants in this group of genes [19, 28, 29, 30]. Furthermore, other specialists may be useful by the geneMDD for particular cases. For instance, *NKX2–1* molecular defects are often linked to thyroid and neurological disorders, necessitating specific expertise [31]. Despite the recommendations of the ACMG, which aim to standardise variants interpretation, our study highlights the critical role of involving molecular geneticists specialised in respiratory diseases. Most of the reclassified variants were variants identified by non‐French genetic laboratories with more recent expertise on genes like *RTEL1*. The geneMDD reclassified the pathogenicity of 4 genetic variants on the basis of new scientific evidence available or specific genotype/phenotype correlation results: out of 65 variants initially classified as pathogenic or likely pathogenic, one (1.5%) was reclassified as VUS and one (1.5%) as likely benign. Additionally, out of the 17 VUS, one (5.9%) was reclassified as likely pathogenic and another (5.9%) as pathogenic.

Regarding pulmonary and extra‐thoracic diseases, the gene MDD confirmed all diagnoses. Furthermore, we observed a higher proportion of unclassifiable lung fibrosis (33%) compared to what is reported in the literature [32]. This can be explained by cases coming from tertiary‐level centres with an expertise in ILD before any genetic analysis.

In our study, geneMDD modified the therapy for 30 patients (30%) and lower age, a non‐IPF ILD diagnosis, and the presence of SRG and TRG variants were associated with therapeutic change recommendation. Regarding therapeutic interventions, evidence is very limited as no trial specifically designed for patients with inherited lung fibrosis offers evidence‐based support for therapeutic decisions [13]. Post hoc analysis of the ASCEND and CAPACITY trials revealed that pirfenidone slows the decline of lung function in patients with a TRG variant [33]. A retrospective study suggested that pirfenidone and nintedanib showed a beneficial effect on lung function decline in patients with a TRG variant [34]. Several retrospective studies suggested a more rapid decline in FVC and a particular toxicity of immunosuppressants in patients with short telomeres or with a TRG variation [11, 35, 36, 37]. Despite their limitations, these data probably influenced the prescription of antifibrotics and the discontinuation of immunosuppressants in patients with TRG variants, particularly in those with unclassifiable fibrosis. The pathophysiology of pulmonary fibrosis in SRG variant carriers is probably different, including reticulum endoplasmic stress, with possible efficacy of immunomodulatory therapy [38]. Our study confirms the recent retrospective study by Zhang et al. showing the influence of telomere length on their antifibrotic prescription [39].

Although the data currently available do not make it possible to propose a truly targeted treatment, several in vitro, retrospective or prospective studies justify proposing and analysing treatments specifically targeting variants: elexacaftor/tezacaftor/ivacaftor for *ABCA3* deficiency, methionine for *MARS1* deficiency, or Janus Kinase Inhibitor for *COPA* and *STING1* related syndromes [40, 41, 42]. It is experts who make these decisions in a multidisciplinary discussion, that the data are collected and reported so as to finally be able to propose prospective studies in the near future [43].

In total, since September 2021, the geneMDD proposed WGS for 20 families, but so far, all results have been negative for the four families who have already been analysed. These results are comparable to those of WGS in a cohort of familial pulmonary fibrosis with only 25% of the families “genetically explained” [11]. However, the WGS analyses for diagnostic purposes focused on genes with known functions. The pipeline was not fully adapted to the discovery of new genes, nor to multifactorial diseases. Thus, the current rate of negative genetic results with the targeted NGS method is unlikely to be improved by WGS at this time. However, the practical and economic benefits of WGS compared to targeted NGS should be analysed, especially after reanalysing existing data without resequencing. Moreover, the data generated by WGS can be reanalysed as knowledge and bioinformatics progress.

Our study had several limitations. The geneMDD relies on the referring physicians to volunteer to discuss their patient, inducing a selection bias. Additionally, we do not have data from a comparison group to evaluate therapeutic changes prior to the implementation of the geneMDD. Moreover, the patients discussed came from centres with a high level of expertise in the field of ILD, and our findings may not be extrapolated to centres less specialised in ILD. In addition, we did not assess the impact of geneMDD on the prognosis of the patients (data not shown) [44, 45].

Our study emphasises the importance of multidisciplinary discussion in the management of patients with familial or genetic ILD. The collaboration among professionals in the discussion is crucial for integrating genetic data with diagnostic information, leading to a more precise guidance of patient treatment, recommendations for any subsequent investigations, and a clear follow‐up plan for both patients and their relatives. For these reasons, we believe that a geneMDD might become the standard of care for patients with suspected or confirmed genetic ILD. Further prospective studies are necessary to assess the impact of geneMDD on the natural history and prognosis of these patients.

## Author Contributions

**Giovanni Franco:** conceptualization (equal), data curation (equal), formal analysis (equal), investigation (equal), methodology (equal), writing – original draft (equal), writing – review and editing (equal). **Ibrahima Ba:** conceptualization (equal), data curation (equal), investigation (equal), writing – original draft (equal), writing – review and editing (equal). **Nadia Nathan:** conceptualization (equal), data curation (equal), investigation (equal), writing – original draft (equal), writing – review and editing (equal). **Cécile Guerin:** data curation (equal), investigation (equal), writing – original draft (equal), writing – review and editing (equal). **Albane Lassus:** data curation (equal), writing – review and editing (equal). **Caroline Kannengiesser:** conceptualization (equal), data curation (equal), investigation (equal), writing – original draft (equal), writing – review and editing (equal). **Antoine Froidure:** conceptualization (equal), data curation (equal), investigation (equal), writing – original draft (equal), writing – review and editing (equal). **Effrosyni Manali:** conceptualization (equal), investigation (equal), writing – original draft (equal), writing – review and editing (equal). **Vincent Bunel:** conceptualization (equal), investigation (equal), writing – review and editing (equal). **Philippe Bonniaud:** data curation (equal), writing – review and editing (equal). **Diane Bouvry:** data curation (equal), writing – review and editing (equal). **Marie Pierre Debray:** conceptualization (equal), investigation (equal), writing – original draft (equal), writing – review and editing (equal). **Pierre Antoine Juge:** conceptualization (equal), data curation (equal), investigation (equal), writing – review and editing (equal). **Ralph Epaud:** data curation (equal), investigation (equal), writing – review and editing (equal). **Camille Louvrier:** data curation (equal), investigation (equal), writing – review and editing (equal). **Aurélie Plessier:** data curation (equal), investigation (equal), writing – review and editing (equal). **Flore Sicre de Fontbrune:** data curation (equal), investigation (equal), writing – review and editing (equal). **Lidwine Wémeau‐Stervinou:** data curation (equal), methodology (equal), writing – review and editing (equal). **Sylvain Marchand Adam:** data curation (equal), investigation (equal), writing – review and editing (equal). **Alexandre Chabrol:** data curation (equal), investigation (equal), writing – review and editing (equal). **Arnaud Maurac:** data curation (equal), investigation (equal), writing – review and editing (equal). **Laurent Savale:** data curation (equal), investigation (equal), writing – review and editing (equal). **David Montani:** data curation (equal), methodology (equal), writing – review and editing (equal). **Caroline Raynal:** data curation (equal), investigation (equal), writing – review and editing (equal). **Marina Konyukh:** data curation (equal), investigation (equal), writing – review and editing (equal). **Arthur Mageau:** conceptualization (equal), data curation (equal), investigation (equal), methodology (equal), writing – original draft (equal), writing – review and editing (equal). **Bruno Crestani:** conceptualization (equal), data curation (equal), investigation (equal), methodology (equal), writing – original draft (equal), writing – review and editing (equal). **Vincent Cottin:** conceptualization (equal), data curation (equal), investigation (equal), methodology (equal), writing – review and editing (equal). **Alix de Becdelièvre:** conceptualization (equal), data curation (equal), investigation (equal), methodology (equal), writing – original draft (equal), writing – review and editing (equal). **Raphaël Borie:** conceptualization (equal), data curation (equal), data curation (equal), investigation (equal), investigation (equal), methodology (equal), methodology (equal), supervision (equal), supervision (equal), writing – original draft (equal), writing – original draft (equal), writing – review and editing (equal), writing – review and editing (equal).

## Ethics Statement

This study was approved by the local ethics committee (CPP Ile de France 1, no. 0811760). The clinical charts of the patients were collected on a standardised and anonymous form. All patients signed informed consent for genetic analysis, including for research purposes.

## Conflicts of Interest

E.M. reports payment or honoraria for lectures, presentations, speaker bureaus, manuscript writing, or educational events from AstraZeneca, Boehringer Ingelheim, Demo, Roche, Elpen HELLAS; support for attending meetings and/or travel from Boehringer Ingelheim and Elpen HELLAS, all outside the submitted work. P.B. reports grants or contracts from AstraZeneca; payment or honoraria for lectures, presentations, speaker bureaus, manuscript writing, or educational events from Sanofi, AstraZeneca, GSK; support for attending meetings and/or travel from AstraZeneca, Novartis, Sanofi, Boehringer Ingelheim, Stallergene, and GSK; participation on a Data Safety Monitoring Board or Advisory Board for AstraZeneca, Novartis, Sanofi, GSK, and Boehringer Ingelheim. b.c. reports grants or contracts from Boehringer Ingelheim; consulting fees from BMS, Boehringer Ingelheim, Chiesi, GSK, and Sanofi; payment or honoraria for lectures, presentations, speaker bureaus, manuscript writing, or educational events from Astra Zeneca, BMS, Boehringer Ingelheim, GSK, Novartis, Roche, and Sanofi; support for attending meetings and/or travel from Astra Zeneca, BMS, Boehringer Ingelheim, Roche, and Sanofi; participation on a Data Safety Monitoring Board or Advisory Board for BMS, Boehringer Ingelheim, Horizon, and Sanofi; and he is president of the board of the Fondation du Souffle, a French charity, all outside the submitted work. R.B. reports consulting fees from Boehringer Ingelheim, Ferrer, and Sanofi; payment or honoraria for lectures, presentations, speaker bureaus, manuscript writing, or educational events from Boehringer Ingelheim; support for attending meetings and/or travel from Boehringer Ingelheim, all outside the submitted work. The other authors declare no conflicts of interest.

## Supporting information


**Table S1.** Pulmonary diseases, HRCT and histology pattern.

## Data Availability

The data that support the findings of this study are available from the corresponding author upon reasonable request.
