# Supplementary material for: Impact of A Multidisciplinary Team Discussion for Genetic Lung Fibrosis
Source: Respirology. 2025 Mar 26;30(6):523–32. doi: 10.1111/resp.70039 (PMC12128731; doi:10.1111/resp.70039)
Supplement: Supplementary file 1 — Table S1. Pulmonary diseases, HRCT and histology pattern. [file RESP-30-523-s001.docx]

**Supplementary Table**. Pulmonary diseases, HRCT and histology pattern

| **Pulmonary diseases** | |
| --- | --- |
| Patient with pulmonary diseases | 103 (89.6%) |
| Unclassifiable ILD, n (%) | 38 (33.0%) |
| IPF, n (%) | 37 (32.2%) |
| NSIP, n (%) | 11 (9.6%) |
| PPFE, n (%) | 6 (5.2%) |
| cHP, n (%) | 3 (2.6%) |
| DIP, n (%) | 3 (2.6%) |
| Alveolar proteinosis, n (%) | 2 (1.7%) |
| Emphysema, n (%) | 1 (0.9%) |
| Squamous cell carcinoma, n (%) | 1 (0.9%) |
| Alveolar microlithiasis, n (%) | 1 (0.9%) |
| **HRCT pattern** | |
| Patient who performed HRCT | 107 (93.0%) |
| Indeterminate for UIP | 44 (41.1%) |
| Definite or probable UIP | 32 (29.9%) |
| Alternative diagnosis | 26 (24.3%) |
| Not ILD | 5 (4.7%) |
| **Histology pattern** | |
| Patient who performed biopsy | 26 (22.6%) |
| UIP | 12 (46.2%) |
| Indeterminate | 6 (23.1%) |
| Alveolar proteinosis | 2 (7.7%) |
| NSIP | 2 (7.7%) |
| PPFE | 2 (7.7%) |
| Squamous cell carcinoma | 1 (3.8%) |
| Not ILD | 1 (3.8%) |

Data are n(%) HRCT and histological pattern proposed at geneMDD ILD: interstitial lung diseases; IPF: idiopathic pulmonary fibrosis; NSIP: non-specific interstitial pneumonia; PPFE: pleuro-parenchymal fibroelastosis; HP: hypersensitivity pneumonitis; DIP: desquamative interstitial pneumonia; UIP: usual interstitial pneumonia
